# Increasing the qualitative understanding of optimal functionality in older adults: a focus group based study

**DOI:** 10.1186/s12877-016-0244-z

**Published:** 2016-03-23

**Authors:** Samal Algilani, Lina Östlund-Lagerström, Ida Schoultz, Robert J. Brummer, Annica Kihlgren

**Affiliations:** Nutrition and Physical Activity Research Centre, School of Health and Medical Sciences, Örebro University, S-701 82 Örebro, Sweden; Nutrition Gut Brain Interactions Research Centre, School of Health and Medical Sciences, Örebro University, S-701 82 Örebro, Sweden

**Keywords:** Older adults, Optimal functionality, Person Centered Care, Focus group discussions

## Abstract

**Background:**

Decreased independence and loss of functional ability are issues regarded as inevitably connected to old age. This ageism may have negative influences on older adults’ beliefs about aging, making it difficult for them to focus on their current ability to maintain a good health. It is therefore important to change focus towards promoting Optimal Functionality (OF). OF is a concept putting the older adult’s perspective on health and function in focus, however, the concept is still under development. Hence, the aim was to extend the concept of optimal functionality in various groups of older adults.

**Methods:**

A qualitative study was conducted based on focus group discussions (FGD). In total 6 FGDs were performed, including 37 older adults from three different groups: group 1) senior athletes, group 2) free living older adults, group 3) older adults living in senior living homes. All data was transcribed verbatim and analyzed following the process of deductive content analysis.

**Results:**

The principal outcome of the analysis was “to function as optimally as you possibly can”, which was perceived as the core of the concept. Further, the concept of OF was described as multifactorial and several new factors could be added to the original model of OF. Additionally the findings of the study support that all three cornerstones comprising OF have to occur simultaneously in order for the older adult to function as optimal as possible.

**Conclusions:**

OF is a multifaceted and subjective concept, which should be individually defined by the older adult. This study further makes evident that older adults as a group are heterogeneous in terms of their preferences and views on health and should thus be approached as such in the health care setting. Therefore it is important to promote an individualized approach as a base when caring for older adults.

**Electronic supplementary material:**

The online version of this article (doi:10.1186/s12877-016-0244-z) contains supplementary material, which is available to authorized users.

## Background

Between the years of 2000 and 2050 it is estimated that older adults over the age of 60 will increase from 605 million to 2 billion worldwide [1]. Decreased independence and loss of functional ability are issues regarded as inevitably connected to old age. This ageism [2] may have negative influences on older adults’ beliefs about aging, hindering them from focusing on the available resources to maintain mental [3], social [4] and physical well-being [5–7]. Because of the steadily growing population of aged individuals and these existing dogmas, it is of importance to steer away from such negatives beliefs, directing focus towards increasing perceived well-being and promoting optimal functionality [8].

In the context of ageing it is of importance to emphasize that the population of older adults is not a homogeneous group. It is, on the contrary, heterogeneous, including individuals of a wide age-range with varying degrees of health [9], different culture, attitudes and practices [10]. Lopez et al. [11] states that assessing the older adult’s own preferences is essential in order to promote health and personal satisfaction. In addition, older adults, independent of context, wish to be engaged when it comes to their health and care [12–14]. Therefore, viewing older adults as a homogeneous group, overlooking their differences, may hinder the possibility of an individualized health care; introducing personalized health care is thus an urgent matter.

Optimal Functionality (OF) was recently explored by our research group as a new concept to identify the older adult’s individual experience of well-being in different areas of life. OF can be seen as a concept adopting a holistic approach to older adults health constituting three major cornerstones: body-related factors (e.g. physical well-being), self-related factors (e.g. mental well-being) and external factors (e.g. environmental factors); the concept is under development and our previous study identified a lack in the qualitative understanding of OF [8]. The concept of OF focuses on the preferences of the aged individual and could thus be a tool to promote individualized health care and increase the subjective well-being of older adults. The concept is, however, still in need of further development. Hence, the present study aimed to extend the qualitative knowledge of the concept of optimal functionality by focus group discussions performed in various groups of older adults.

## Methods

### Study design

This study had a descriptive design with the qualitative approach of content analysis inspired by Hsieh & Shannon ‘s directed approach [15]. Focus group discussion (FGD) was chosen as a data collection method, in order to expand the knowledge about the concept of OF. According to Kitzinger [16], the aim of the FGD is to explore, discover, and clarify the opinions and views of individuals in a way not possible with one-on-one interviews. Further, FGDs are preferentially used when the objective is to expand knowledge based on qualitative data in order to answer questions relating to an already existing concept [17].

The study was approved by the Uppsala Regional Ethics Review Board (dnr. 2012/309) and written informed consent was obtained from all study participants.

### Selection criteria

Older adults (≥65 years), living in a city in central Sweden, were invited to participate in the study (see Table 1). The older adults invited were selected from an already existing cohort of older adults, constituting a large number of individuals who previously had given their consent to participate in interviews and discussions about health and well-being. The cohort of older adults included: 1) senior athletes (SA), i.e. older adults actively competing in the sport of orienteering, 2) a group of free-living older adults (FL), i.e. living in ordinary housing without assistance from health care staff and 3) a group of older adults living in senior living homes (SLH) and receiving assistance from health care staff. Hereafter the three groups of older adults will be referred to as SA (senior athletes), FL (free-living) and SLH (senior living home).

**Table 1:** Overview of the three different groups of older adults

Group (Age: mean ± SD)	Housing situation	Need for support/services in daily life	Physical activity	Health index score* (median (range))
SA (Age: 71.5 ± 4.2) (Sex: 7 women, 7 men)	Free living	Manage daily life by themselves	Actively practicing and competing in orienteering, i.e. cross-country running	31.5 (22–36)
FL (Age: 75.2 ± 7.2) (Sex: 5 women, 6 men)	Free living	Manage daily life, some have meals on wheels	Engage in everyday physical activity such as walking outdoors, cycling, swimming	29 (21–34)
SLH (Age: 86.7 ± 3.9) (Sex: 12 women)	Senior living homes	Need assistance to manage daily life, all have home care services	Engage in basic physical activity such as walking to joint dining hall, walking in corridors, and moving in the apartment	28 (22–36)

*Health Index is a self-completed questionnaire of functional health, asking nine questions related to vigor, temper, fatigue, loneliness, sleep, dizziness, bowel function, pain and mobility. The score ranges between 9 and 36, where higher scores are favorable and indicates good health [36]

The FGD participants were recruited as follows:

1. An information meeting for the SA was held at Örebro University. Out of the 30 attending the meeting, 14 senior athletes gave their permission to participate in the FGD.
2. The participants in the FL group were selected from an existing cohort of 257 older adults. Every fifth person on the list received an information letter for participation in the FGD. Eleven people consented to participate in the study.
3. An information meeting was held for the SLH and the older adults were asked to participate in this study. Out of 30, 12 older adults accepted to participate in the FGD.

In all three groups of older adults, there was variation in age, sex, and living situation, as shown in Table 1. The three groups of older adults had all retired from work and had a living situation corresponding to their needs and wishes.

### Data collection

#### Developing the interview guide

The three suggested cornerstones of OF, namely body-related factors, self-related factors and external factors [8] were used as a framework to construct questions for a semi-structured discussion guide. This guide was adopted with the intention of not overly controlling the discussions [18]. The questions asked were: 1) *What makes you experience OF overall?* 2) *What makes you experience OF due to body-related factors?* 3) *What makes you experience OF due to self-related factors?* 4) *What makes you experience OF due to external factors?* Follow-up questions such as: *Can you clarify what you mean by that?* or *Can you elaborate on that please?* were also included. Since the term OF might be difficult for the older individuals to interpret, it was additionally described as “what makes you experience well-being, health and function at the best level currently possible for you”.

#### The FGDs

In order to adhere to the recommended number of FGD participants per group [16], the older adults from groups SA, FL and SLH were divided into 2 groups, respectively, generating total of 6 FGD groups, Hence, a total of 6 FGDs were coordinated and carried out during the spring of 2014 in accordance with the procedure suggested by Kitzinger [16]. The size of the groups ranged from 5 to 8 participants, with a total of 37 participants.

All discussions lasted from 45 to 90 min depending on response saturation i.e. when no new information arose the FGD was terminated. In accordance to McLafferty [19], when no new information is added to the data collection saturation has been obtained. The two FGDs with the SA and the two FGDs with the FL were carried out at Örebro University. The two FGDs with the SLH were organized at the senior living home. The FGDs with groups SA and FL were led by the first author (PhD-student, Registered Nurse (RN)) as moderator and the second author (PhD-student) as assisting observer. All authors, except for RJB (Professor, Medical Doctor), were involved with the FGDs for the third group, but the third author (PhD) and last author (Professor, RN) mainly led them. Every session began with giving information about the objective of the study, and the participants were informed about the confidentiality of the collected data.

### Data analysis

All discussions were audio recorded and transcribed verbatim. The first and second author read the transcripts thoroughly and made an effort to interpret the data as a whole. The data was subsequently analyzed following the process of deductive content analysis, as described by Elo & Kyngäs [20]. Moreover, a structured analysis matrix was developed (see Table 2) and new subcategories, belonging to one of the former main categories, were chosen from the text and coded according to these categories. A complete overview of the FGD analysis and results are presented as Additional files (1–6). The current structure of the concept OF [8] was used as a model for testing the coded categories [20] throughout the analytical process. Further, there was a continuous dialogue amongst the co-authors to validate the analyzed data [21]. Throughout the whole process of data analysis peer scrutiny [22] was adopted, e.g. via co-assessment by researchers in the research group, presentation at a scientific meeting and discussion in a multi-professional seminar session. In the results section, quotations are presented to verify the findings; all quotations were translated from Swedish to English by a bilingual (English and Swedish) translator originating from the U.S.

**Table 2:** An example of coding the data to the categorization matrix

	Body-related factors	Self-related factors	External factors
What makes older adults experience OF?	- Healthy food	- Gratitude for life	- Social activities
	- Independence	- Experience new things	- Family
	- Maintenance of physical ability	- Acts of kindness	- Modern technology

### Consolidated criteria for reporting qualitative studies (COREQ)

In accordance with BioMed Central editorial policies the qualitative methods and the reporting of their results adhere to the COREQ guidelines [23]. A complete COREQ checklist has been uploaded as Additional file 7.

## Results

The qualitative content analysis generated several new subcategories that could be added to the original model of OF (presented as “newly added factors” in Table 3) [8], presented below in italics. Quotes are presented in bold italics and between quotation marks.

**Table 3:** Overview of newly added factors to the concept of OF

	Aspects	Newly added factors	Discussed by group^a^
Body-related factors	Health Aspects	Free from disease	3
		Gut health	2,3
		Healthy food	1,2,3
		Relaxation	2
		Physical function	2
		Annual health check	1
	Activity Aspects	Maintenance of physical ability	2
		To occupy one self	2
		Physical exertion	1
	Autonomy Aspects	Being able to move	3
		Staying independent	1,2
		Maintaining daily routines	1,2
Self-related factors	Adjustment Aspects	Gratitude for life	2,3
		Being content	1,2
		Living in the presence	1
		Becoming dependent	2
	Capability Aspects	Experience new thing	1,2,3
		Engaging activities	1,2,3
		Experiencing nature	1,2,3
		Engaging activities	1,2,3
		Enjoying tasty food	1,2,3
		Acts of kindness	2,3
		Positive attitude	1,2
		Enjoying life	1,2
		Having goals	1,2
	Mental Aspects^b^	---	---
External factors	Social Aspects	Social activities	1,2,3
		Social network	1,2
		Family/significant others	1,2
	Environmental	Thriving at home	1,2,3
		Health care	1,3
		Assistive tools	3
		Modern technology	1,2
		Silence	1

^a^1 = SA; 2 = FL; 3 = SLH

^b^The topic of Mental Aspects was not discussed by any of the FGDs

### Body-related factors

When talking about OF in relation to the body, all older adults were unanimous about the fact that one’s bodily function is of great importance in order to experience OF. Although all groups described physical health as being important, they tended to discuss it in relation to their current health status such as being *free from disease* and *having annual health checks*.

***“But there is also something about just doing fun things…Enjoying doing things…Then you feel much healthier.”****(SA)*

***The most important thing is to maintain health, that you are able to do what you are supposed to do [everyday tasks]…so that you can manage things.”****(FL)*

***To be healthy and feel well…to be free from disease and misery.”****(SLH)*

Furthermore, all groups agreed on the importance of eating *healthy food,* and *gut health* was also perceived as important, as discussed by the FL and SLH groups.

The FL group, but not the other two groups, also mentioned the importance of *maintaining physical ability/function* and *occupying ones self (keeping busy).* Also, the three groups of older adults interpreted physical activity in different ways, based on their functional abilities, e.g. the SA, uniquely stated *physical exertion* as a prerequisite for experiencing OF.

***“When you have exerted yourself, when it has gone well, you feel really good afterwards.”*** (SA)

All the older adults felt that engaging in physical activity was essential in order to experience OF, i.e. to feel that you are functioning as optimally as possible. However, *relaxation* was discussed as equally important.

***“It is very important for me to relax. It is the direct opposite of running. I need that [to relax] as well.”*** (SA)

Autonomy was seen as essential to all older adults; the older adults in group SLH spoke about it on the level of *being able to move*, while the other two groups talked about *staying independent* and *maintaining daily routines*.

***“…the most important thing is to maintain health. That you are able to do what you are supposed to do, especially when you live alone…Four rooms and a kitchen in a two-story house, a garden, and a garage. Yes, managing the yard and the garden and then doing what I want to do.”*** (FL)

***“As long as you can get up in the morning and get dressed and things like that…You may be a little light-headed or something, but as long as you are on your feet, you are happy.”*** (SLH)

### Self-related factors

In relation to the self, the older adults discussed OF as *being content* and satisfied with life, having the capability to *engage in different activities*, and being able to look forward to things in life, i.e. events such as social activities, trips or a good meal. The older adults spoke of the importance of the self to “function” optimally and also stated that it is as important for experiencing OF as the function of the body.

All three groups talked about the importance of adjustment to be able to function as optimally as possible, but on different levels, e.g. the older adults in group SLH talked about replacing one activity with another in order to actively adjust to everyday life.

***“I read and do crossword puzzles a lot…What else should I do when I can’t cross stitch anymore…my neck and shoulders won’t cooperate so [therefore] I do crossword puzzles and read books.”*** (SLH)

The two other groups spoke of active adjustment as acceptance of *becoming dependent* of others and the importance of making it comfortable for you in old age. To actively adjust oneself to everyday life by *practicing gratefulness* was discussed by all groups. *Being content* (with life) was also something that was viewed as important by the SA and FL groups. When talking about active adjustment, the older adults in SA and FL mentioned that it was more than just adjusting to life and *being grateful*; they also discussed the importance of *living in the present*.

***“You have to enjoy life as long as you are healthy.”*** (SA)

***“I live in the presence and I don’t look back.”*** (FL)

Being capable of *engaging in activities* was important to all three groups, e.g. taking part in cultural activities, having the capability to *experience new things*, *experiencing nature*, and *enjoying tasty food* were essential in order to function optimally. Also, having the capability to carry out *acts of kindness, having goals*, looking forward to things, and *enjoying life* were also seen as essential for the two latter groups.

None of the groups mentioned anything at all about elements or factors regarding mental health.

### External factors

External factors and elements were perceived as essential and just as important as body-related factors and self-related factors in functioning as optimally as possible, according to the older adults. The ambient environment of the older adult, comprised of things such as social activities and network, housing situation, modern technology, and good quality health care were external elements that were spoken of in relation to OF.

All of the older adults perceived socializing as something essential in order to function as optimally as possible on all levels. All three groups spoke of *social activity* and social interaction as essential factors for OF, e.g. enjoying mealtimes with others. The older adults in groups SA and FL also talked about the importance of having a *social network* and being important to others in order to function as optimally as possible.

***“Oh how good that I can still do lots of things and bring joy to and help my kids.”*** (SA)

***“You have to get out and meet people for as long as you possibly can.”*** (FL)

***“To sit, drink coffee and talk.”*** (SLH)

The groups SA and FL spoke of the importance of *family* and saw it as crucial, while the older adults in group SLH did not discuss the subject.

When the older adults spoke of the importance of the environment in order to function as optimally as possible, they also discussed the importance of *thriving in your own home* and being content with your housing situation. The third group of older adults discussed having *assistive tools* to make everyday life easier and to be able to function independently. The older adults in groups SA and FL discussed *modern technology* as being important.

***“We take advantage of the current technology; we have a greater chance…of living longer.”*** (SA)

***“The tablet computer thinks like my brain…the tablet does exactly what I want.”*** (FL)

Accessible, high quality *health care* was discussed by the older adults in all groups as being crucial in order to experience OF. The group SA also talked about the importance of experiencing *silence* and perceived it as something essential in order to function optimally.

## Discussion

The older adults included in this study identified the core meaning of OF as equal to “functioning as optimally as you possibly can”, i.e. to be at your present best in your everyday life. Also, the older adults in this study, described the cornerstones of OF (body-related factors, self-related factors and external factors) as closely linked with one another, all three with inevitable contribution to the well-being of the older adult. Moreover, the older adults described OF as having a wide range of different meanings, especially since the concept reflects individual preferences. Further, our results indicate that OF is highly subjective and thus needs to be approached in a subjective manner, supporting the suggestions made in the previous paper from our group [8]. The current study also resulted in the description of several factors that were new to the concept, see Table 3, as compared to our previous model [8].

This study further shows that OF is based on the older adults’ own preferences and is dependent on body-related, self-related and external factors. All of the older adults involved in the current study, independent of age and health status, had various thoughts and experiences on what they believed was necessary in order to function as optimally as possible. Differences, as well as similarities, in thoughts and experiences could be found both between and within the three groups. Cline [24] has previously emphazised the heterogenocity in the aged population.

The topic of physical activity, already known as a factor in the concept of OF, was discussed in all groups, yet on somewhat different levels. The older adults in group SLH spoke of moving about and taking walks in the corridors at the senior living home they lived in, while the older adults in group FL discussed the importance of physical activity in everyday life, such as taking the stairs instead of the elevator or cycling instead of driving or taking the bus. In SA, however, the older adults were very physically active and spoke about physical exertion and the joy of challenging themselves when it came to physical activity. This might indicate that older adults learn to adjust to decreased physical ability, as demonstrated by the fact that the older adults residing in senior living homes are content with short walks, while the SA feel the need to push themselves physically. Further, the importance of adjusting to everyday life, e.g. replacing an unmanagable activity with a managable one and acceptance of becoming dependent on others, was spoken of in all groups. The older adults in group SLH talked about making active and conscious choices to be able to adjust to everyday life, e.g. trading needlework for crossword puzzles. The older adults from groups SA and FL instead talked about what might come in the future, how you need to be prepared to make adjustments in order to have a meaningful life, even with limited function.

Another interesting finding was related to food, which was perceived as very important by all three groups in that it is a necessity for health, it provides a positive experience in life, and it plays a big role in social activities. This demonstrates that food contributes to all three cornerstones of OF. Food being essential for health was spoken of by all groups, but food as a positive experience and as a social activity was only discussed by the SA. However, this should be interpeted with caution, since it could be an effect of the FGD methodology where the topics develop in relation to the individuals present and the interplay between them. It is also possible that this topic was influenced by the three groups’ living situations and opportunities for social interaction, e.g. the SA group might have the ability to “go out” more. The importance of food and mealtime setting for older adults has been previously described [25, 26]. Furthermore, experiencing gut health was discussed as an important factor for optimal functionality, however, in contrast to previous research from our group this was not discussed by the SA [27].

Moreover, using assistive tools and internet communication technology (ICT) tools that enhance everyday life was perceived as important for all older adults. In SLH, the older adults mostly discussed assistive tools in the sense that they made everyday life easier, while the other two groups spoke of ICT-tools as being something fun to use.

The older adults included in this study expressed various individual preferences based on their own functional abilities and health status. This notion should be especially important within the health care context, because, when failing to involve the individual in her or his own care, aspects of the older adult’s life that are of great importance to the health care outcome can be overlooked. Person Centered Care (PCC) is an approach that is used within the health care to enable an individulized approach to the older adult’s care. Attributes such as holistic, individualized, respectful and empowering can be used by health care professionals in order to set the foundation for a person-centered approach [28]. In order to aid person-centered care there is a need for the older adult to function as optimally as they can in the health care context, i.e. thus OF can be used to facilitate a person-centered approach. Thórarinsdóttir and Kristjánsson [29] confirm this by stating that the patient’s experiences, values and needs are significant for person-centered participation. Nolan [30] further emphasizes that it is of importance to have in mind the person’s individual variations and subjective interpretations, but to also be aware of the heterogeneity among different groups of people, such as patients and health care professionals. The concept of OF could be of assistance in this matter, identifying important aspects for the older individual to increase overall well-being and health. For example, rehabilitation of older adults and transitions from hospital to home, or from home to a longterm care facility, would probably be more successful when considering all areas of importance for the older adults’ optimal function. We suggest that the concept of OF in the future could function as a tool to increase the awareness of health care personnel of areas of particular importance to the older individual’s perceived health and well-being. Pin-pointing those areas, and facilitating the older adult’s perceived function within those areas, might increase the experience of health, and ease the adjustments in everyday life on the basis of the older adult’s own abilities and preferences.

Despite mental health being one of the nine aspects that originally surfaced as being important for experiencing OF [8], none of the older adults in this study spoke of mental health. This is an interesting finding and there may be several reasons for the lack of discussion on this topic. Perhaps the older adults felt ashamed talking about this sensitive topic. One American study, including Korean American older adults, found a negative attitude toward mental health services among this group and they perceived depression as a sign of weakness. Furthermore, the older adults believed that having a family member with a mental illness brought shame to the whole family [31]. Other studies on mental health attitudes showed that older adults living in rural areas had a negative attitude toward seeking help for mental illness [32, 33]. Discussing mental health is of significance in the sense that research clearly shows that there are feelings of shame connected to it. Therefore, we call for further research to explore what older adults experience in relation to mental aspects and OF in order to fully cover all nine aspects that constitute the concept of OF.

### Methodological considerations

This study has an evident strength in its originality, as it is the first to describe OF among older adults from a qualitative perspective. There are, however also some limitations to this study that should be considered and discussed. It can be argued that the FGD method is a limitation due to not receiving enough depth in the data [19]. However, the method of using FGDs is effective for collecting data when the research aims to seek opinions and beliefs [34], which supports the aim of this study. Bringing insight to the opinions and beliefs of older adults, in relation to the concept of OF, was preferred in this study in order to further develop and expand the understanding of the concept. Yet, generlisations of qualitative interpretations from FGD data should be approached with some caution since the topics discussed develop in relation to the individuals’ present and the interplay between them. Also, the qualitative data obtained in this study was analysed with a deductive approach, as it was judged as the best fit to the research question, yet this might limit the chance of gaining new input on the structure of the concept of OF. However, this approach is commonly used when testing concepts, categories, models or hyptheses [35] as was the case in the current study.

The aspect of mental health was not spoken of in any of the FGDs. This could be a methodological issue due to feelings of shame when discussing mental health in a group setting. However, there are different opinions on this matter. In a paper by Jayasekara [34] it is pointed out that some researchers have the opinion that FGDs are not useful for discussing sensitive topics. On the contrary, other researchers have the exact opposite experience of conducting FGDs, meaning that FGDs are successful because they make it possible for participants to share their experiences and beliefs in a safe environment [34].

## Conclusions

In this qualitative focus group-based study, the concept of OF was further explored and developed. The older adults described the core of OF as “functioning as optimally as you can”, i.e. “to be at your present best”. Moreover, the OF model, as previously described by Algilani et al. [8] was supported, and several new factors relating to the concept were found and may in the future be added to the original structure of the OF model.

The three cornerstones of OF were described as closely linked with one another, all three with inevitable contribution to the well-being of the older adult. The concept was further described as subjective, since it is based on individual preferences.

However, the study showed lack of discussion regarding mental aspects, which was one of the nine aspects comprising the original model of OF. Therefore, further research on this particular topic is needed. We propose that future studies should conduct individual interviews with older adults in order to discuss the meaning of mental aspects in relation to OF. The same methodology could also be of use to further deepen the understanding of the concept at a total.

## Availability of data and materials

The Additional files 1, 2, 3, 4, 5 and 6 present a complete overview of the FGD analysis from all 6 focus group discussions. Additional file 7 presents the COREQ checklist.

## Additional files

Additional file 1: Complete overview of the FGD analysis, results from SLH group 1 (PDF 82 kb) Additional file 2: Complete overview of the FGD analysis, results from SLH group 2 (PDF 88 kb) Additional file 3: Complete overview of the FGD analysis, results from FL group 1. (PDF 104 kb) Additional file 4: Complete overview of the FGD analysis, results from FL group 2. (PDF 98 kb) Additional file 5: Complete overview of the FGD analysis, results from SA group 1. (PDF 98 kb) Additional file 6: Complete overview of the FGD analysis, results from SA group 2. (PDF 94 kb) Additional file 7: Consolidated criteria for reporting qualitative studies (COREQ): 32- (PDF 144 kb)
