# Supplementary material for: Increasing the qualitative understanding of optimal functionality in older adults: a focus group based study
Source: BMC Geriatr. 2016 Mar 23;16:70. doi: 10.1186/s12877-016-0244-z (PMC4806442; doi:10.1186/s12877-016-0244-z)
Supplement: Additional file 1: — Complete overview of the FGD analysis, results from SLH group 1 (PDF 82 kb) [file 12877_2016_244_MOESM1_ESM.pdf]

Additional file 1. Complete overview of the FGD analysis, results from SLH group 1

Main categories	Generic categories	Sub-categories	Meaning Units
**Body-related factors**	Activity Aspects	Physical Activity	*Move about at home*
			Gymnastics on Fridays feels good
			Important to move about
	Autonomy Aspects	Being able to move (new)	*Being able to move*
			Getting up in the morning
			Being able to walk outdoors
			It's good to move about
	Health Aspects	Free from disease (new)	*To be healthy*
			Being free from disease and misery
			Being free from ailments
		Gut Health	*Important to have good bowel movement*
			If you move about (walking) bowel movement will be good
		Healthy food (new)	*Nutritious food*
			Food is of importance
		Subjective Health	*To feel well/healthy*
**Self-related factors**	Adjustment Aspects	Gratitude (new)	*Gratitude for having an attendant*
			Feeling spared (from misery)
		Tasty food (new)	*To have tasty food*
**External Factors**	Demographic Aspects	Economy	*To be able to afford (an attendant)*
	Environmental Aspects	Assisted care (new)	*Getting lots of help from a nurse*
			Happy to have an attendant
			Receiving help in the dining hall if needed
		Assistive tools (new)	*Amenities that have arrived and improved health*
			Assistive tools in the homes
		Thriving at home (new)	*A very good place to live*
