# Supplementary material for: Increasing the qualitative understanding of optimal functionality in older adults: a focus group based study
Source: BMC Geriatr. 2016 Mar 23;16:70. doi: 10.1186/s12877-016-0244-z (PMC4806442; doi:10.1186/s12877-016-0244-z)
Supplement: Additional file 2: — Complete overview of the FGD analysis, results from SLH group 2 (PDF 88 kb) [file 12877_2016_244_MOESM2_ESM.pdf]

Additional file 2. Complete overview of the FGD analysis, results from SLH group 2

Main categories	Generic categories	Sub-categories	Meaning Units
**Body-related factors**	Activity Aspects	Physical Activity	*Walking in the corridors*
			Good to move about
			Keep up (moving) as well as you can
			Walk a bit everyday/daily
	Health Aspects	Being able to move (new)	*As long as you can get up in the morning*
			To be able to dress (your self)
		Healthy food (new)	*We eat "real" food*
			Always eat on a regular basis (food)
**Self-related factors**	Adjustment Aspects	Active adjustment (new)	*To solve crossword puzzles and read book when you no longer are able to do needlework*
	Capability Aspects	Acts of kindness (new)	*Funds*
			Do some work (charity)
		Cultural Activity (new)	*To watch TV when it is sports*
			Listening to music is nice
			To listen to rock-music
			Solving cross-word puzzles
			Reading books
			You get what you need (right here) instead of going somewhere else
**External Factors**	Environmental Aspects	Accessible health care	*They walk outside together with those who cannot do it by themselves*
		Assisted care	*Having the "walker" to hold on to*
		Assistive tools (new)	*The medical care here is extremely good*
		High quality medical care	*Everything is really good (health-care personnel, dining hall)*
		Thriving at home (new)	*Solgården is the best among senior living homes)*
			To sit, drink coffee and talk
	Social Aspects	Social activity	*Walking in the corridors and meeting people*
			Entertainment and coffee-drinking
