# Supplementary material for: Increasing the qualitative understanding of optimal functionality in older adults: a focus group based study
Source: BMC Geriatr. 2016 Mar 23;16:70. doi: 10.1186/s12877-016-0244-z (PMC4806442; doi:10.1186/s12877-016-0244-z)
Supplement: Additional file 3: — Complete overview of the FGD analysis, results from FL group 1. (PDF 104 kb) [file 12877_2016_244_MOESM3_ESM.pdf]

Additional file 3. Complete overview of the FGD analysis, results from FL group 1

Main categories	Generic categories	Sub-categories	Meaning Units
**Body-related factors**	Activity Aspects	Maintaining Physical Ability (new)	*As long as I can move about and nothing prevents that*
	Autonomy Aspects	Dependence (new)	*We become dependent of other people's services in order to feel well*
	Health Aspects	Function	*Good enough balance*
		Gut Health (new)	*To maintain/keep stomach and bowels in good condition*
		Maintenance of daily routines (new)	*Pull apart the blinds*
		Physical Activity	*Dancing is fun (as physical activity)*
		Staying independent	*Important to take care of your self for as long as possible*
		Subjective Health	*It's all well if you get to be healthy/well*
		To relax (new)	*To lie down on the bed- it's nice*
**Self-related factors**	Adjustment Aspects	Active adjustment	*Adjustment to/for needing help*
		(Independence)	*To manage everything that has to be done (when you live alone/ by your self)*
			To be able to do what I wish/want to do
			Gymnastics for seniors
			The bicycle is a blessing
			To be physically active
			To go outside and walk
			To move about
			To ride a bike/cycle
			To take a walk
			To take care of your garden and do gardening
			To take out your bicycle
			To work out at a gym
			To be able to bring in the newspaper
			To be able to get up in the morning
			To be able to go down the stairs
			To be able to let the cat outside
			To be able to make coffee
			It is disabling to loose you ability to read (impaired vision)
			Stiffness/rigidity
			Takes longer time to warm up the muscles
			The balance is not on top
			The musculature turns/gets stiff
			Vision is very important in order to see and read
			Maintaining your health
			When I'm ill, I'm not happy
			To rest and pause/take a break
			Wonderful to just lie still/be still
			You have to rest in between (turns)
			Difficult not to manage everyday life by your self
			To learn that you are dependent of others
			You have to adjust to your age
			You have to make it comfortable for your self

	Capability Aspects	Act of kindness (new)	*I like helping others*
			It is easier to help people and be there for them
			To do a good deed
			To help others is uplifting
			To help out with homework
			To work as volunteer gives satisfaction
		Awareness by external events (new)	*Satisfaction to immerse your self into nature shows on TV*
			To step outside your self for a while
			To turn out side yourself
		Cultural activity (new)	*Go to the movies/cinema, lectures*
			It is an experience to see Flashdance (the show)
			Listen to music
			Music
			Reading books
			The most important things is to read
			Watch TV that provides (knowledge) e.g. Discovery Channel
		Inner resources	*Engage in things*
			Get out and do things whatever the weather
			Keeping the brain active by cross word puzzles/Sudoku
		Learning new things	*To learn things*
			To have the desire to learn things
			To be motivated to learn things
		Positive attitude (new)	*To feel happy*
			To be positive
		Spirituality	*A sense of spirituality is important*
			To light a candle in church
		To experience Nature (new)	*To experience the best sides of all seasons*
		To have goals (new)	*To succeed with things you have planned*
	Environmental Aspects	To thrive (new)	*To be wealthy and thrive with (your) life*
**External Factors**	Social Aspects	Family (new)	*Children*
			Family
			Friends
			Grandchildren
			Relatives
			The happiness of having children and grandchildren
		Interacting with younger people	*I feel good when I get to be together with young people*
			One of my best moments is when I sit down with a bunch of adolescen (young people) and help them
		Social activities	*Social encounters are important*
			To do things together with other
			To experience things together with someone
			You should/have to get out and mix with people for as long as you can
		Social relations/network (new)	*Good relations with neighbors*
			Good relations are very stimulating
			I enjoy spending time with my neighbors and friends
			You wish/want to be needed

Still being important to others	*To be needed by someone else*
	To help them in need
	To help those that are in worse condition
