# Supplementary material for: Increasing the qualitative understanding of optimal functionality in older adults: a focus group based study
Source: BMC Geriatr. 2016 Mar 23;16:70. doi: 10.1186/s12877-016-0244-z (PMC4806442; doi:10.1186/s12877-016-0244-z)
Supplement: Additional file 4: — Complete overview of the FGD analysis, results from FL group 2. (PDF 98 kb) [file 12877_2016_244_MOESM4_ESM.pdf]

Additional file 4. Complete overview of the FGD analysis, results from FL group 2

Main categories	Generic categories	Sub-categories	Meaning Units
**Body-related factors**	Activity Aspects	Physical Activity	*Go out for a bike ride*
			I am physically active/I move about
			I do gymnastics
			Kept up the exercise
			To keep up
			To move about/ to exercise
			To work in garden
		To occupy one self (new)	*To have something to do*
			You occupy your self all day
		Physical function (new)	*The body does what you want it to do*
			To be able to do what you want
	Health Aspects	Gut health (new)	*To feel that the stomach works properly*
			That the stomach feels fine
		Healthy food (new)	*Eating the right things*
			Healthy food
			Keeping the blood sugar on a reasonable level
		Subjective health	*No ailments*
			No being in any kind of pain
			To feel healthy
**Self-related factors**	Capability Aspects	Being content (new)	*To be content with life*
			To be content with your self
		Cultural activities (new)	*I read quite a lot*
			Music
			Nice to get away (travel)
			Solving cross word puzzles
			To attend a show/performance of some kind
		Curiosity for others (new)	*To be curious about other people*
		Engaging in lustful activities (new)	*Genealogy research*
			Have a fun hobby
			Hunting
			Just do fun things
			Made a lot of bird feeders
			Own a dog
			To do something that you perceive as fun
			Wild life management
		Enjoying life	*To enjoy*
			To enjoy doing things makes you feel healthier
		Gratitude	*Grateful for each day you are able to get out of bed*
			Life is good
			To feel grateful
		Positive attitude (new)	*Be nice to others*
			Be nice to your self
			Be positive

			No to get annoyed/irritated by other people
			Smile to people makes you happy
			To be able to forgive your self and others
		The ability to look forward to things	*Have something to look forward to*
			To think ahead
			What you have to look forward to
		To experience nature (new)	*I love the forest*
			Nature
			The sun
			To feel united with nature
			To get the feeling that you are one with all of it (nature)
			To walk around in the forest and picking mushrooms
**External Factors**	Demographic Aspects	Economy	*To manage well economically*
	Environmental Aspects	Modern technology (new)	*It has endless possibilities (the tablet)*
			One of these iPhones any idiot can (manage), it's enough just to look at it
			Smartphone is easy (to use)
			The smart tablet does what I want it to do
			The smart tablet thinks as my brain
			To be positive about modern technology
			To see the possibilities (with modern technology)
			You can do so much with a smart phone
		Thriving at home (new)	*Fun/feels good to come home (from trips)*
	Social Aspects	Family (new)	*Having a god family relationship*
			To feel good with the people that are around you
			To play with the grandchildren
			You (rather) want to do something with them (children and grandchildren)
			You have good friends and neighbors
		Feeling safe with your network (new)	*To feel safe by having contacts*
			To get help if you need it (by social network)
		Social network	*A lot of fun to go out with friends*
			Coworkers
			The importance of social (network)
			The social network is quite important
			To manage well socially
