# Supplementary material for: Increasing the qualitative understanding of optimal functionality in older adults: a focus group based study
Source: BMC Geriatr. 2016 Mar 23;16:70. doi: 10.1186/s12877-016-0244-z (PMC4806442; doi:10.1186/s12877-016-0244-z)
Supplement: Additional file 5: — Complete overview of the FGD analysis, results from SA group 1. (PDF 98 kb) [file 12877_2016_244_MOESM5_ESM.pdf]

Additional file 5. Complete overview of the FGD analysis, results from SA group 1

Main categories	Generic categories	Sub-categories	Meaning Units
**Body-related factors**	Activity Aspects	Physical Activity	*Go for a morning walk*
			Out running in the woods
			To be able to use your legs
			To be able to walk
			To be able to walk and run
			To move about
			To move about is good
			When running my quick round (it feels good)
			Wonderful to walk, jog a little
		Physical exertion (new)	*Feels good when you've made an effort*
			The satisfaction that comes when you've made an effort
			You push (the body) as much as you can
	Autonomy Aspects	Maintaining daily routines (new)	*To be able to do you usual things*
			To get out of bed in the morning
		Staying independent (new)	*As long as I'm healthy I want to be in charge of my self*
			To be able to make your own decision
	Health Aspects	Healthy food (new)	*To eat a lot of vegetables*
			To eat wisely (more vegetables)
		Sleep (new)	*To lie down for a moment everyday*
			To sleep several hours at a stretch makes one happy
		Subjective health	*To be healthy*
			To feel healthy and strong
		Water	*To drink water is of importance*
**Self-related factors**	Adjustment Aspects	Appreciate everyday life (new)	*You learn to appreciate everyday thing*
			You learn to appreciate the small things
		Being content (new)	*Being content with your day*
			If you have done what you could it is good
			To be content
		To accept one self (new)	*To accept that you are who you are and that there will be changes*
			To accept that you don't manage (physically) as much as before
	Capability Aspects	Act of kindness (new)	*Nice to get out for lunch once in a while*
			Satisfactory to help others to succeed
			Stimulating to help others
			To do things together in groups give satisfaction
			To help kids in the forest (with maps) is fun
		Cultural activity (new)	*Gets happy when listening to jazz*
			Listening to classical music
			Music is for the soul
			To do oil painting
			To go o the theatre
			To rock to music makes you happy
		Enjoying tasty food (new)	*Being able to enjoy tasty food with a glass of wine*
		Silence (new)	*To hear the silence*

			Silence makes you feel well
		To experience nature (new)	*Feels very good when out in nature*
			The fragrance of the forest
			To be outdoors
			To be outdoors in nature
			To be outdoors in the forest/woods in the summer and in the winter
			To get away from the noise in the city
			To hear chirping of birds
			To listen to the birds
			When it blooms
			Wonderful to listen to the Blackbird
		To have goals (new)	*Satisfaction when you have a goal and when you reach it*
			There is a drive
		To live in the presence (new)	*To live today and not look back*
		To SEE nature (new)	*A delight to the eye when you see a nature scene*
			Soothing to watch the sunset
			To see the colors (in the nature)
**External Factors**	Environmental Aspects	Accessible health care (new)	*Health care and doctors*
		Being content with your housing situation (new)	*Environment/surroundings is important*
			It is really good where I live
			The housing situation means a lot
			To be grateful for your housing situation
		Modern Technology (new)	*There are both upsides and downsides with modern technology*
			We benefit from the rapid development of technology, it increases our chances to survive
	Social Aspects	Being important to others	*Satisfactory to know that you will be missed if you are not there*
			To receive satisfaction from others
			You feel that you are needed
		Family (new)	*Fun to see the grandchildren*
			Positive to have family, children, and grandchildren
			To hug your grandchildren
		Food (new)	*It always tastes better to eat together with someone*
			Tasty food is a social matter
		Social activities (new)	*Be involved in social interactions*
			Enjoy meeting people
			Go out together
			It's never dull to spend time with (these) friends
			Spend time with young orienteers
			The social part of meeting people is very important
			To talk to people
			You receive more and more friends
