# Supplementary material for: Increasing the qualitative understanding of optimal functionality in older adults: a focus group based study
Source: BMC Geriatr. 2016 Mar 23;16:70. doi: 10.1186/s12877-016-0244-z (PMC4806442; doi:10.1186/s12877-016-0244-z)
Supplement: Additional file 6: — Complete overview of the FGD analysis, results from SA group 2. (PDF 94 kb) [file 12877_2016_244_MOESM6_ESM.pdf]

Additional file 6. Complete overview of the FGD analysis, results from SA group 2

Main categories	Generic categories	Sub-categories	Meaning Units
**Body-related factors**	Activity Aspects	Physical Activity	*Cross-country skiing*
			Exercise
			Hiking in Kilsbergen if the weather is right
			Nice to bicycle along the rivers
			Take a walk everyday before breakfast
			To be out on the golf course for a few hours
			To find control points in the forest
			To move about
			To take walks in the forest
			You have to keep moving about
	Health Aspects	Annual health check	*Attend to annual health check*
		Healthy food (new)	*Eat as healthy as possible*
			You have to eat right
		Not to be stressed (new)	*Important to not be stressed*
		Subjective health	*To be healthy*
**Self-related factors**	Adjustment Aspects	To have time (new)	*To have time to do things*
			To own your time
	Capability Aspects	Cultural Activity (new)	*Beautiful music*
			Culture
			Dancing
			Music
			Nice trips
			Read books
			Take classes
			Tasty food
			To attend a concert
			To be involved in a non-profit organization
			To travel
			Watch a movie
		Engaging in lustful activities (new)	*Do things that are perceived as fun*
			Engage in things that are pleasurable
		Experience new things (new)	*Do things that you haven't done before*
			Experience new things
			Experience new places
		Positive attitude (new)	*To possess a positive attitude (towards life)*
		To be committed (new)	*To have a commitment (to things?)*
		"To create" food (new)	*Cooking is a lot of fun*
			Satisfactory when you are able to grow your own food
		To enjoy the presence (new)	*Enjoy for as long as you can*
			Enjoy the moment
			Take pleasure in the little things
			Take the opportunity and enjoy for as long as you are healthy
		To experience nature (new)	*Be fond of nature*

			Be outdoors in daylight
			Get outdoors and just enjoy your self
			Listen to birdsong
			Satisfactory when you have done something good
			Sit outdoors and have a coffee
			To be outdoors a lot
			Walk about in the forest/woods
			Walk around in the forest and pick berries
		To have goals (new)	*To have a goal*
		To SEE nature (new)	*It's the nicest thing in the spring when you see the greenery*
**External Factors**	Environmental Aspects	Thriving at home (new)	*Delightful to be at home*
			Nice to be at home
			To thrive where you live
			We have a covered terrace that I love
	Social Aspects	Family (new)	*Children*
			Grandchildren
			To be together with the grandchildren
		Social relations (new)	*Feels nice at home*
			Good to have contacts
			Nice friends
