# Supplementary material for: Increasing the qualitative understanding of optimal functionality in older adults: a focus group based study
Source: BMC Geriatr. 2016 Mar 23;16:70. doi: 10.1186/s12877-016-0244-z (PMC4806442; doi:10.1186/s12877-016-0244-z)
Supplement: Additional file 7: — Consolidated criteria for reporting qualitative studies (COREQ): 32- (PDF 144 kb) [file 12877_2016_244_MOESM7_ESM.pdf]

Additional file 7. Consolidated criteria for reporting qualitative studies (COREQ): 32-item checklist

Manuscript: Increasing the qualitative understanding of optimal functionality in older adults: a focus group based study

Samal Algilani[1], Lina Östlund-Lagerström[1,2], Ida Schoultz[1,2] , Robert J. Brummer[1,2], Annica Kihlgren[1]

[1]Nutrition and Physical Activity Research Centre and [2]Nutrition Gut Brain Interactions Research Centre, School of Health and Medical Sciences, Örebro University, S-701 82, Örebro, Sweden

Consolidated criteria for reporting qualitative studies (COREQ): 32-item checklist

Developed from:
Tong A, Sainsbury P, Craig J. Consolidated criteria for reporting qualitative research (COREQ): a 32-item checklist for interviews and focus groups. *International Journal for Quality in Health Care*. 2007. Volume 19, Number 6: pp. 349 – 357

No. Item	Guide questions/description	Response
**Domain 1: Research team and reflexivity**		
Personal Characteristics		
1. Inter viewer/facilitator	Which author/s conducted the interview or focus group?	Samal Algilani (SA), Lina Östlund-Lagerström (LÖL), Ida Schoultz (IS), Annica Kihlgren (AK).
2. Credentials	What were the researcher's credentials? E.g. PhD, MD	SA: PhD-student, registered nurse (RN), LÖL: PhD-student, MSc sport science, IS: PhD, AK: Professor, (RN), Robert J. Brummer: Professor, MD.
3. Occupation	What was their occupation at the time of the study?	AS: PhD-student, LÖL: PhD-student, IS: Assistant professor, AK: Professor, Robert J. Brummer: Professor, MD.
4. Gender	Was the researcher male or female?	Four were female, 1 was male.
5. Experience and training	What experience or training did the researcher have?	SA: MSc in Psychiatric Nursing, RN<br>LÖL: MSc in Sport Science

		IS: PhD in Biomedicine<br>AK: Prof. in Care Science, has performed multiple FGD and interview studies,<br>Robert J. Brummer: Prof. in Medicine, MD (gastroenterology).
Relationship with participants		
6. Relationship established	Was a relationship established prior to study commencement?	The older adults invited to participate in this study were selected from an already existing cohort of older adults, available within the Nutrition and Physical Activity research Centre at Örebro University. The cohort constituted a large number of older individuals who previously had given their consent to participate in interviews and discussions about health and well-being.
7. Participant knowledge of the interviewer	What did the participants know about the researcher? e.g. personal goals, reasons for doing the research	The participants were introduced to the researchers and informed about the study purpose and design via information meetings or an informational letter, see manuscript in the method section/ *selection criteria*.
8. Interviewer characteristics	What characteristics were reported about the interviewer/facilitator? e.g. Bias, assumptions, reasons and interests in the research topic	The FGD moderators introduced themselves at the beginning of each FGD, by name, occupation and educational status. The participants were briefly introduced to the purpose of our research by the presentation of the results from our previously published paper on the topic of optimal functionality, see reference no. 8.

**Domain 2: study design**		
Theoretical framework		
9. Methodological orientation and Theory	What methodological orientation was stated to underpin the study? e.g. grounded theory, discourse analysis, ethnography, phenomenology, content analysis	See manuscript in the method section/ *study design* and *data analysis*.
Participant selection		
10. Sampling	How were participants selected? e.g. purposive, convenience, consecutive, snowball	See manuscript in the method section/ *selection criteria.*
11. Method of approach	How were participants approached? e.g. face-to-face, telephone, mail, email	See manuscript in the method section/ *selection criteria.*

12. Sample size	How many participants were in the study?	See manuscript in the method section/ *selection criteria.*
13. Non-participation	How many people refused to participate or dropped out? Reasons?	See manuscript in the method section/ *selection criteria.*
Setting		
14. Setting of data collection	Where was the data collected? e.g. home, clinic, workplace	See manuscript in the method section/ *Data collection - The FGDs.* .
15. Presence of non-participants	Was anyone else present besides the participants and researchers?	No.
16. Description of sample	What are the important characteristics of the sample? e.g. demographic data, date	See manuscript in the method section/ *selection criteria* and Table 1.
Data collection		
17. Interview guide	Were questions, prompts, guides provided by the authors? Was it pilot tested?	See manuscript in the method section/*data collection – Developing the interview guide.*
18. Repeat interviews	Were repeat interview carried out? If yes, how many?	No.
19. Audio/visual recording	Did the research use audio or visual recording to collect the data?	See manuscript in the method section/*data analysis.*
20. Field notes	Were field notes made during and/or after the interview or focus group?	No.
21. Duration	What was the duration of the interviews or focus group?	See manuscript in the method section/ *data collection – The FGDs.*
22. Data saturation	Was data saturation discussed?	See manuscript in the method section/ *data collection – The FGDs.*
23. Transcripts returned	Were transcripts returned to participants for comment and/or correction?	No.
**Domain 3: analysis and findings**		
Data analysis		

24. Number of data coders	How many data coders coded the data?	Two, SA and LÖL.
25. Description of the coding tree	Did authors provide a description of the coding tree?	Yes, a description was provided to the coauthors.
26. Derivation of themes	Were themes identified in advance or derived from the data?	See manuscript in the method section/ *data analysis.*
27. Software	What software, if applicable, was used to manage the data?	No software was used.
28. Participant checking	Did participants provide feedback on the findings?	No.
Reporting		
29. Quotations presented	Were participant quotations presented to illustrate the themes/findings? Was each quotation identified? e.g. participant number	See manuscript in the result section.
30. Data and findings consistent	Was there consistency between the data presented and the findings?	Yes.
31. Clarity of major themes	Were major themes clearly presented in the findings?	Yes.
32. Clarity of minor themes	Is there a description of diverse cases or discussion of minor themes?	Yes, the FGD findings are thoroughly presented in the Additional files 1-6 and diversions are presented in the result section and the discussion of the manuscript.
